# The Transient IFN Response and the Delay of Adaptive Immunity Feature the Severity of COVID-19

**DOI:** 10.3389/fimmu.2021.816745

**Published:** 2022-01-14

**Authors:** Gang Xu, Furong Qi, Haiyan Wang, Yu Liu, Xin Wang, Rongrong Zou, Jing Yuan, Xuejiao Liao, Yang Liu, Shuye Zhang, Zheng Zhang

**Affiliations:** ^1^ Institute for Hepatology, National Clinical Research Center for Infectious Disease, Shenzhen Third People’s Hospital, The Second Affiliated Hospital, School of Medicine, Southern University of Science and Technology, Shenzhen, China; ^2^ Department for Infectious Diseases, Shenzhen Third People’s Hospital, Shenzhen, China; ^3^ Shanghai Public Health Clinical Center, Fudan University, Shanghai, China; ^4^ Shenzhen Research Center for Communicable Disease Diagnosis and Treatment of Chinese Academy of Medical Science, Shenzhen, China; ^5^ Guangdong Key Laboratory for Anti-Infection Drug Quality Evaluation, Shenzhen, China

**Keywords:** COVID-19, ScRNA-seq, early immune feature, IFN response, delayed adaptive immunity

## Abstract

COVID-19 patients show heterogeneous and dynamic immune features which determine the clinical outcome. Here, we built a single-cell RNA sequencing (scRNA-seq) dataset for dissecting these complicated immune responses through a longitudinal survey of COVID-19 patients with various categories of outcomes. The data reveals a highly fluctuating peripheral immune landscape in severe COVID-19, whereas the one in asymptomatic/mild COVID-19 is relatively steady. Then, the perturbed immune landscape in peripheral blood returned to normal state in those recovered from severe COVID-19. Importantly, the imbalance of the excessively strong innate immune response and delayed adaptive immunity in the early stage of viral infection accelerates the progression of the disease, indicated by a transient strong IFN response and weak T/B-cell specific response. The proportion of abnormal monocytes appeared early and rose further throughout the severe disease. Our data indicate that a dynamic immune landscape is associated with the progression and recovery of severe COVID-19, and have provided multiple immune biomarkers for early warning of severe COVID-19.

## Introduction

SARS-CoV-2 infection causes COVID-19 with different severity. Most patients develop only mild symptoms, while a minor fraction develop severe diseases, especially for the elderly with pre-existing conditions (1). Immunological perturbations are associated with COVID-19 severity, including increased immature myeloid suppressor cells (2, 3), T cell depletion (4), and cytokine storm (5, 6). Thus, the successful or impaired immune responses were acknowledged playing crucial roles. Previous studies suggest that IFN response (7), T cell response (8), and potential antibody-dependent enhancement (ADE) (9) are potential factors, causing subsequent deterioration of coronavirus induced diseases. However, the reported roles of these immune elements in the pathogenesis of severe COVID-19 are often inconsistent, e.g., both heightened or impaired IFN responses in severe COVID-19 were reported (10, 11). There are also inconsistent reports of anti-viral CD4^+^ T-cell (12, 13), CD8^+^ T-cell responses (14), and humoral immune responses (12, 15) in patients with different COVID-19 severity. One important cause of those discrepancies is likely due to the heterogenous nature of COVID-19 and its dynamic clinical course (16). Indeed, a COVID-19 patient may show largely different immune responses at different stages of the disease (17). Thus, mechanistic understanding of the COVID-19 pathogenesis will require a thorough understanding of the entire dynamic processes.

There were several datasets investigating non-synchronized COVID-19 samples collected primarily at peak level severity or convalescence (18–20). However, the dataset from samples taken longitudinally at an earlier stage of infection (prior to the development of serious diseases) is still absent. One mystery with COVID-19 is that patients can quickly deteriorate without any warning. Understanding such triggering events and identifying potential prediction factors may lead to more effective measures to prevent disease deterioration. However, the stressed medical system during the COVID-19 pandemic usually looks after the sickest patients first, so information/data collected prior to disease deterioration are scarce. To this end, by benefiting from strict contact tracing, quarantine measures and designated hospitalization in Shenzhen, China, we were able to study a valuable cohort of COVID-19 patients by closely following their clinical courses.

Here, we presented such a scRNA-seq dataset of peripheral immune cells in SARS-CoV-2 infected patients, containing longitudinal samples of COVID-19 patients with asymptomatic, mild, and severe diseases. This critical resource provides a great opportunity to decipher the pivotal immunological events preceding the development or resolving of the SARS-CoV-2 induced diseases. Evidence pointed to a highly dynamic circulating immune landscape, namely, remodeling of myeloid and lymphoid compartments matching with the development and recovery of severe COVID-19. In addition, our data highlighted the early immunological events that precede the stage for subsequent development of severe COVID-19. Understanding these mechanisms is the holy grail for the COVID-19 study.

## Results

### Clinical Characteristics in a Closely Monitored Cohort of Patients With COVID-19 With Varying Severity

**Figure 1:**
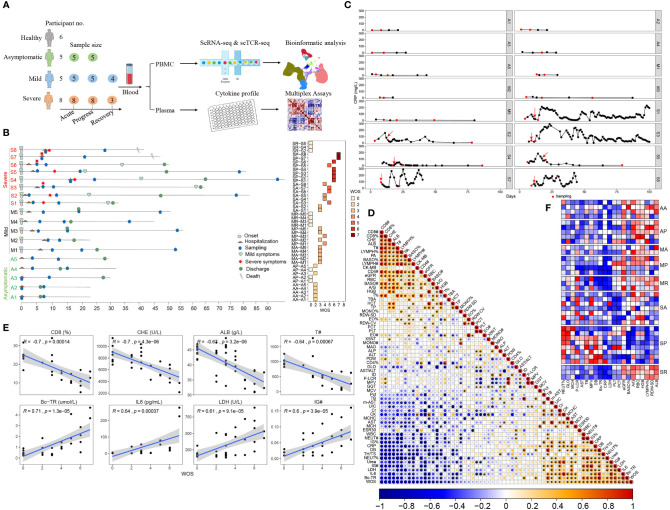
Research scheme and clinical characteristics of the studied COVID-19 patients. **(A)** Graphical overview of the study. Blood from 5 asymptomatic, 5 mildly and 8 severely ill COVID-19 patients and 6 healthy controls were collected for scRNA-seq and cytokine profiling analysis. **(B)** Timeline of each studied COVID-19 patient enrolled in this study. Critical points indicated are hospitalization, sampling, severity turning point, discharge and death date as days after symptom onset (left). The assessment COVID-19 severity is described in the *Materials and Methods*. The right panel shows the disease severity at each sampling date according to the WHO ordinal scale (WOS). In total, 43 samples from COVID-19 patients were collected and comprised of 8 groups, namely, AA, AP, MA, MP, MR, SA, SP, and SR. **(C)** Representative dynamic monitoring of CRP levels from selected COVID-19 patients, namely, 5 asymptomatic, 4 mildly and 7 severely ill patients. The red arrow indicates when the disease becomes serious, and the red dot indicates when PBMC was sampled. **(D)** Correlation matrix of the 63 clinical parameters from the 18 studied COVID-19 patients. The bottom bar corresponds to the absolute value of the Spearman Rank correlation coefficient (*P-value < 0.05). The abbreviated terms are described in the *Materials and Methods*. **(E)** The top 4 positively and 4 negatively WOS-correlated clinical parameters, (# represents cell count). **(F)** The heatmap shows the available relative levels of WOS-correlated clinical parameters near each individual sampling, according to the 8 studied COVID-19 groups.

To identify the characteristics of the early immune response that led to the variable severity of COVID-19, we performed single cell RNA-seq of 49 PBMC samples from five asymptomatically infected, five mildly, and eight severely ill COVID-19 patients, plus 6 healthy controls (**Figure 1A** and **Table S1**). In particular, among 8 severe COVID-19 cases, conditions of 7 deteriorated after hospitalization, while another one (S7) deteriorated the same day of admission. Two severe COVID-19 cases (S7 and S8) succumbed while 6 recovered. Asymptomatic and mild COVID-19 cases had shorter duration of hospitalization and were discharged within one month (**Figure 1B**). We closely monitored clinical parameters and collected PBMCs at different stages of clinical course, as indicated in **Figure 1B**. For patients with severe COVID-19, PBMCs were collected before, during, and after disease deterioration. The first sampling (Severe Acute, SA) was around 1 week post the symptom onset, the second sampling (Severe Progression, SP) was around 17 days post the symptom onset, and the last sampling (Severe Recovery, SR) was around one month after the discharge. We collected PBMCs from mildly ill patients at times matching with those in severe COVID-19, as the MA (Mild Acute), MP (Mild Progression) and MR (Mild Recovery) groups. For asymptomatic COVID-19 cases, we collected their PBMCs shortly after their admission and one week afterwards, as the AA (Asymptomatic Acute) and AP (Asymptomatic Progression) group (**Figure 1B** and **Table S1**). Accordingly, the eight groups of COVID-19 patients exhibited varying disease severity using the WHO ordinal scale (WOS): Asymptomatic patients scored 0–2; mildly ill COVID-19 patients scored 0–4; while the severely ill patients scored 3–5 in SA, then 5–7 in SP and returned to 0 in SR (**Figure 1B**, right panel). The dynamic clinical courses are also reflected by monitoring individual parameters, such as CRP levels, which are close to normal range in asymptomatic, mildly ill and recovered patients, but increased and fluctuated in severely ill patients (**Figure 1C**).

Correlation analysis revealed the associations between disease severity and clinical parameters, assessing WOS scores and all clinical data from this cohort. We identified previously known factors, such as IL6, LDH, the neutrophils percentages, CD4^+^/CD8^+^ ratio, CRP (positively correlated with WOS) and CD8^+^ T cell percentages, T cell count, lymphocyte count and percentages, CD4^+^ T cell count (negatively correlated with WOS). Besides, we also identified previously unidentified correlations including true bound bilirubin (BC-TR), direct bilirubin (DB), immature granulocytes count and percentage (IG# and IG%), urea (positively correlated with WOS), and cholinesterase (CHE), albumin (ALB), Prealbumin (PA), basophils count (BASO#) and heart-type creatine kinase (CK-MB) (negatively correlated with WOS) (**Figures 1D, E**).

Examining disease severity associated clinical parameters among the eight studied groups, SP clearly stood out, manifested by the highest levels of neutrophils percentages (NEUT%), globulin (GLO), mean corpuscular hemoglobin (MCH), platelet-larger cell ratio (P-LCR), glutamic oxaloacetic transaminase (AST), total bilirubin (TB), mean platelet volume (MPV), direct bilirubin (DB), and BC-TR and lowest levels of estimated glomerular filtration rate (eGFR), BASO counts, the ratio of Albumin/globulin (A/G), red blood cell count (RBC), hemoglobin (HGB), lymphocyte percentages (LYMPH%), standard deviation of red blood cell distribution width (RDW-SD), and ALB, compared with other groups (**Figure 1F** and **Table S2**), whereas those differences are more heterogenous in SA patients, making it difficult to predict disease progression based on clinical parameters. This is also consistent with suddenly worsening COVID-19 in critically ill patients.

### Characterizing the Perturbed Peripheral Immune Cell Landscape in Different Subset of COVID-19 Patients

**Figure 2:**
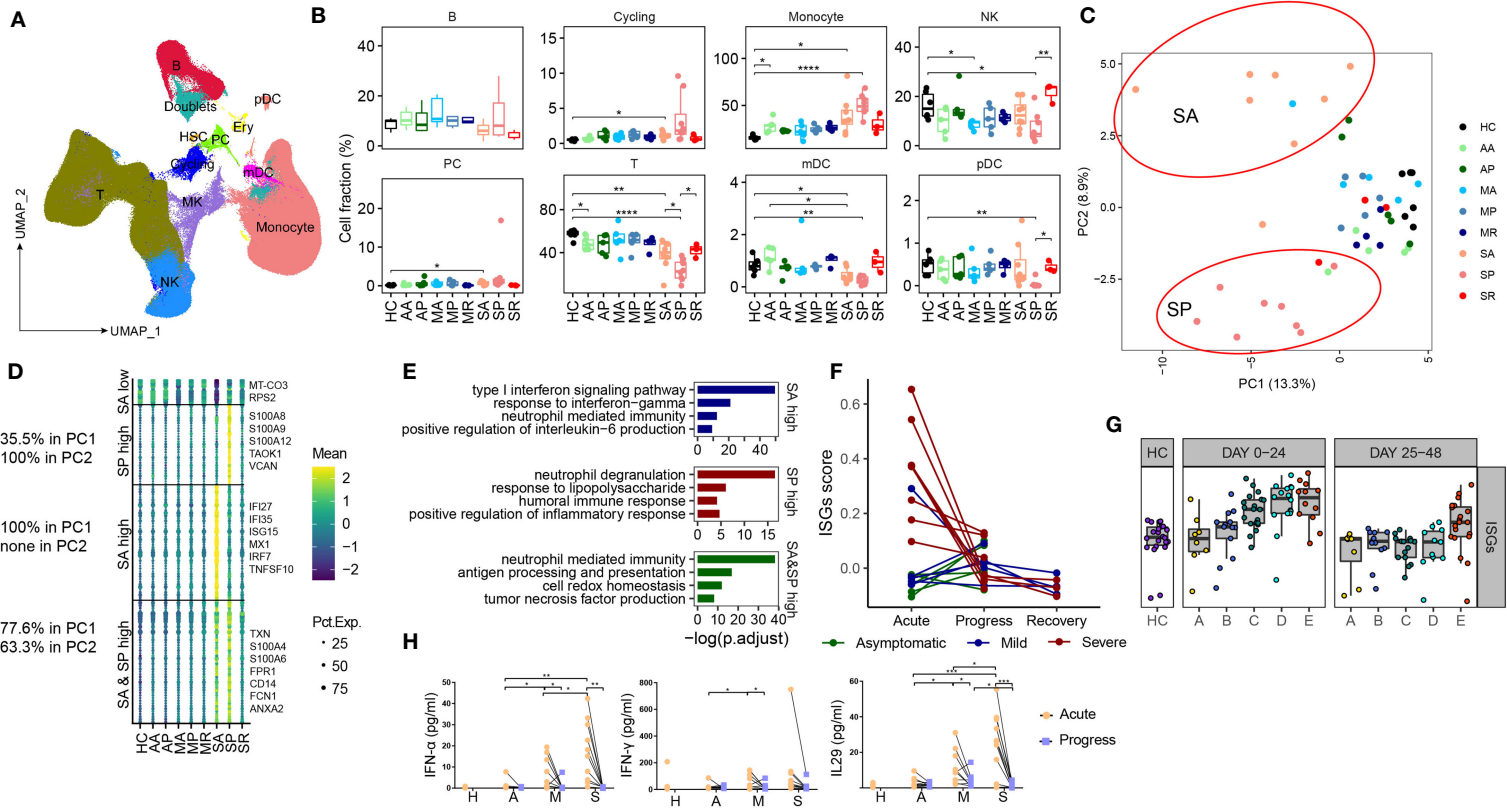
Characterizing peripheral immune perturbations in COVID-19 patients with different clinical course. **(A)** UMAP representation of the ten cell types from the integrated PBMC scRNA-seq dataset (49 samples, 498,151 cells). **(B)** Proportions of various peripheral immune cell types from COVID-19 patients and controls (two-sided Student’s t-test, *P <0.05, **P <0.01, ****P <0.0001). **(C)** Principal-component analysis of averaged transcriptome derived from each individual scRNA-seq data. **(D)** Heatmaps show the SA and SP-specific differentially expressed genes, comprising 4 groups, as higher levels in both SA and SP, higher levels in SA only, higher in SP only, or lower in SA only. Representative genes are indicated at the right side. The percentage of corresponding genes belonging to PC1 and PC2 is indicated at the left side. **(E)** Enrichment of GO biological process (BP) terms for DEGs expressed at higher levels in SA (up), in SP (middle) and in both SA and SP (bottom). **(F)** The scatter plot shows the dynamic changes of the IFN response score of each patient at different time points. **(G)** The scatter plot shows the expression of ISGs in the RNA-seq data of PBMC of 5 groups of COVID-19 patients with different severity levels and healthy controls at 2 time points. **(H)** The plasma levels of IFNα-2a, IFN-γ and IL-29 from the first two samplings of each studied COVID-19 groups and controls, (two-sided Student’s t-test, *P < 0.05, **P < 0.01, ***P < 0.001,****P < 0.0001).

Next, we sought to interrogate immune factors related to different COVID-19 severity by scRNA-seq. A high-quality scRNA-seq dataset composed of 498,151 cells was created and visualized by Uniform Manifold Approximation and Projection (UMAP) projection (**Figure 2A**). The clustering analysis revealed 25 clusters and 10 major cell types annotated by marker genes, namely, T cell (*CD3D*), NK cell (*KLRF1*), B cell (*CD79A*), monocyte (*CD14*, *FCGR3A*), myeloid DCs (mDCs) (*CD1C*), plasmacytoid DCs (pDCs) (*IL3RA*), and plasma cells (PCs) (*IGKC*), megakaryocyte (*MYL9*), cycling cells (*MKI67*), and erythrocytes (*HBB*) (**Figures 2A** and **S1A**). Erythrocytes, megakaryocyte, and doublets were removed in subsequent analysis. Little batch effects were observed (**Figures S1B, C**). The integrated dataset reveals a particularly dynamic immune landscape in patients with severe COVID-19 (changing from SA, to SP, to SR), whereas those in asymptomatic and mild COVID-19 are relatively stable and comparable with controls (**Figures 2B** and **S1D, E**). Consistent with previous reports, proportions of circulating NK cells, T cells, mDCs, and pDCs are significantly decreased in the SP group, while monocyte percentage is significantly expanded. However, in SA and SP group, in whom the severe COVID-19 has yet developed or has recovered, such differences with other COVID-19 groups and controls are subtler (**Figures 2B** and **S1D, E**). Indeed, proportions of NK and pDCs are not significantly reduced in SA, while proportions of NK cells, T cells, mDCs, and pDCs are normalized in SR compared with SP (**Figures 2B** and **S1D**). Another study used RNA-seq to analyze the longitudinal immune response characteristics of a larger cohort of 207 COVID-19 patients, namely, 5 groups of multiple time points, with group A as the asymptomatical patients; group B as the mildly diseased group; group C as the patients admitted to hospital but required no oxygen supplementation; group D as the hospitalized patients need supplemental oxygen and group E as the patients who required assisted ventilation (21). The analysis of this RNA-seq dataset through MarkerBasedDecomposition function in Bisque (22) corroborates the early changes of peripheral immune cells in severe patients (**Figure S1F**). Together, these data indicated that the broad perturbation of blood immune cell compartments closely correlated with development of severe COVID-19 and mainly occurred in SP.

To search for transcriptomic differences between different subsets of COVID-19 patients, we consolidated individual scRNA-seq data as conventional RNA-seq data and performed PCA analysis, and found that PC1 distinguished SA and SP from other groups, and PC2 distinguished SA from SP (**Figure 2C**). The top 100 genes in PC1 and PC2 are listed in **Table S2**. Mapping these genes to UMAP showed that they were mainly derived from myeloid cells (**Figures S1G, H**). The data shows that SA is transcriptomically unique, suggesting that transcriptomic markers from myeloid cells may provide an early warning for developing severe COVID-19. Genes highly expressed in both SA and SP groups, namely, *TXN*, *S100A4*, *S100A6*, *FRP1*, etc., are enriched for neutrophil mediated immunity and antigen processing and presentation pathway; while those highly expressed in SP ar*e S100A8*, *S100A9*, *S10A12*, etc., are involved in neutrophil mediated immunity and response to LPS pathway (**Figures 2D, E**). Notably, those highly expressed in SA include *IFI27*, *IFI35*, *ISG15*, etc., as interferon-stimulating genes (ISGs) (**Figures 2D–F**), indicating a response to high levels of interferon produced *in vivo*. The high expression of ISG in peripheral immune cells of severely ill patients can also be confirmed in the RNA-seq data set (**Figures 2G** and **S1I**). To confirm this, we measured plasma type I, II and III interferon levels from anothor cohort of patients including the different subsets. Indeed, the plasma levels of type I and III IFNs were significantly higher in acute disease stage (AA, MA, SA) versus those with progressive disease (AP, MP, SP), and were also higher in those from SA compared to the AA and MA groups (**Figure 2H**). We confirmed *in vitro* that the lung epithelial cell infected with SARS-CoV-2 induces strong IFN production (**Figure S1J**). Similar reports have been published that SARS-CoV-2 infection stimulates IFN production, which is positively correlated with viral load (23). Together, these data revealed that unique peripheral immune transcriptional signatures emerged both before and during the development of severe COVID-19.

### Remodeling of Myeloid Cell Compartments and Transcriptomes Correlate With the Development of Severe COVID-19

**Figure 3:**
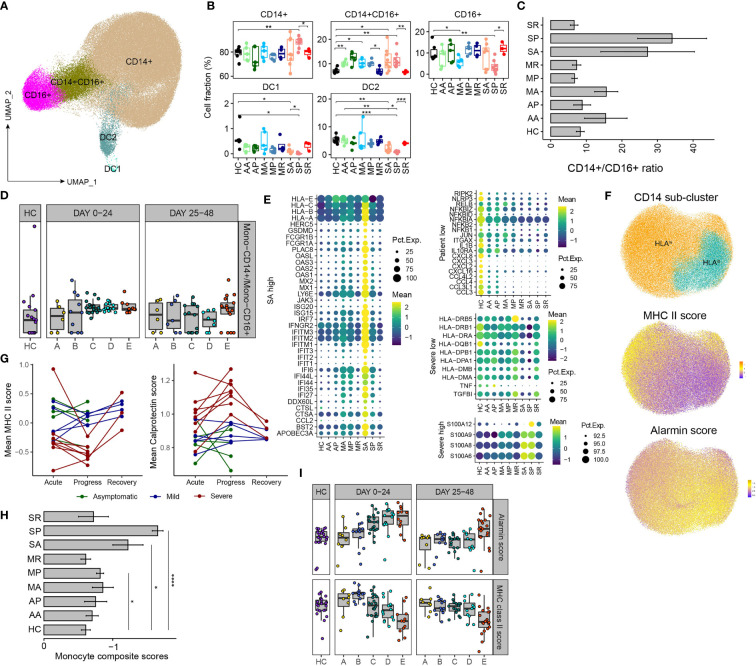
Remodeling of myeloid cell compartment and transcriptional signatures associated with development of severe COVID-19. **(A)** UMAP plot of the major myeloid cell types within PBMCs. **(B)** Proportions of various peripheral myeloid cell types from COVID-19 patients and controls (two-sided Student’s t-test, *P <0.05, **P < 0.01, ***P < 0.001). **(C)** The bar plot of the ratio of CD14^+^/CD16^+^ monocytes. **(D)** The scatter plot shows the ratio of CD14^+^/CD16^+^ monocytes of the 5 groups of COVID-19 patients and healthy controls based on the Marker Based Decomposition analysis of the RNA-seq data in the 2 time zones. **(E)** The heatmaps show the selected differentially expressed genes in CD14^+^ monocyte from comparisons between COVID-19 patients and controls. “SA high” highlights the genes of higher levels in SA; “Patient low” as the genes of lower levels in COVID-19 patients; “Severer low” and “Severe high” as genes of lower or higher levels in both SA and SP. **(F)** UMAP plot of the CD14^+^ monocytes, divided into HLA^high^ and HLA^low^ groups (up). MHC II score (middle) and alarmin score (down) are projected to the UMAP. **(G)** The dynamic changes of the average MHC II score (left) and alarmin score (right) of each patient at different time points. **(H)** The bar plot shows “Monocyte composite scores” across different groups, (two-sided Student’s t-test, *P < 0.05, ****P < 0.0001). **(I)** The dynamic changes of the average MHC II score (bottom) and alarmin score (up) of the 5 groups of COVID-19 patients and healthy controls in the 2 time zones.

Next, we characterized myeloid cell compartment and identified 5 subsets according to the expression of canonical markers: classical monocyte (*CD14*), intermediate monocyte (*CD14*, *FCGR3A*), nonclassical monocytes (*FCGR3A*), DC1 (*CLEA9A*) and DC2 (*CD1C*, *CLEC10A*) (**Figures 3A** and **S2A**). Notably, myeloid compartment underwent dynamic changes before, during and after progression of severe COVID-19. Proportions of DC1 and DC2 significantly decreased in SA than those in controls, reduced further in SP, but normalized in SR, whereas comparable frequencies of DCs were observed between asymptomatic, mild COVID-19 patients and controls. The proportion of CD14^+^CD16^+^ intermediate monocytes increased during acute SARS-CoV-2 infection, and normalized in those recovered. We also noticed increased proportions of CD14^+^ classical monocytes and decreased proportions of CD16^+^ nonclassical monocytes in SP, consistent with early reports by us and others (2, 3, 24), while proportions of classical and nonclassical monocytes were comparable among other studied groups (**Figures 3B** and **S2B–D**). We found association between high ratios of CD14^+^/CD16^+^ monocytes and acute infections, as in AA, MA and SA, while those ratios normalized in recovered patients (AP, MP, MR, and SR), but persistently high CD14^+^/CD16^+^ monocyte ratios were associated with development of severe COVID-19 in SP (**Figure 3C**). Bisque analysis of RNA-seq data of 207 COVID-19 patients also found that severe patients had higher CD14^+^/CD16^+^ monocyte ratios in the periphery at the early stage (**Figure 3D**). Thus, our data revealed that the proportion of CD14^+^ monocytes begins to expand in the early stage of severe patients, and the CD14^+^/CD16^+^ monocyte ratios can serve as an appropriate early prognostic marker for severe COVID-19.

We further characterized the transcriptomic changes of CD14^+^ monocyte from different subsets of COVID-19 patients. Compared with those in controls, the expression levels of genes involved in the innate immune defense were found diminished in CD14^+^ monocytes from COVID-19 patients. The downregulated genes include ones mediating immune signaling, e.g., *RIPK2*, *RLRP3*, and *NFKBID*, etc., and genes encoding cytokine and chemokines, suggesting impaired immune functions of monocytes from COVID-19 patients (**Figure 3E**). The highest expression levels of ISGs is the most prominent feature of CD14^+^ monocytes from SA (**Figure 3E**). Moreover, CD14^+^ monocytes from SA and SP have similar immunosuppressive signature, including downregulation of MHC II genes (*HLA-DRB5*, *HLA-DRB1*, *HLA-DR1*, etc.) and upregulation of alarmin genes (*S100A12*, *S100A9*, *S100A8*, *S100A6*) were observed in both SA and SP, compared with other groups (**Figure 3E**). This is consistent with previous studies that CD14^+^ monocytes from severe COVID-19 patients exhibited signature of immature monocytes, namely, downregulation of MHC II genes and upregulation of alarmin genes (2, 3, 24).

We re-clustered CD14^+^ monocytes into HLA^high^ and HLA^low^ groups, UMAP projection of MHC-II and alarmin signature scores confirmed that HLA^high^ and HLA^low^ CD14^+^ monocytes have a higher MHC-II and alarmin scores respectively (**Figures 3F**). We tightly monitored the MHC-II and alarmin scores at different stages of these 18 patients and found that the SA group showed a higher alarmin score and a lower MHC-II score, which worsened in SP (**Figure 3G**). The differences of “Monocyte composite scores” between different groups are even more apparent (**Figure 3H**). RNA-seq analysis of peripheral immune cells also showed that alarmin expression increased in and MHC-II expression decreased in the early stage of sever COVID-19 patients (**Figures 3I** and **S2E**). Therefore, our data suggest that during acute SARS-CoV-2 infection, the emergence of HLA^low^ population and IFN-response transcriptional signatures in monocytes, likely signify the subsequent progression of severe COVID-19.

### Two Groups of CD8^+^ T Cells With Different Phenotypes and TCR Expansion Associate With Different COVID-19 Severity

To understand the T cell response, we broadly categorized T cells into innate-like T cells (MAIT, NKT, and γδ T) and CD4^+^ and CD8^+^ T cells (**Figure S3A**). A high CD4^+^/CD8^+^ ratio was previously reported in severe COVID-19 (24). We found that the CD4^+^/CD8^+^ ratio started to increase in SA, reached highest levels in SP and normalized in SR (**Figures S3B–D**). Depletion of innate-like T cells is another feature previously reported in severe COVID-19 (25). We found that proportions of innate-like T cells tended to decrease in SA, reached lowest levels in SP and normalized in SR (**Figures S3C, E**). The CD4^+^/CD8^+^ ratio and proportions of innate-like T cells were comparable between non-severe COVID-19 patients and healthy controls.

**Figure 4:**
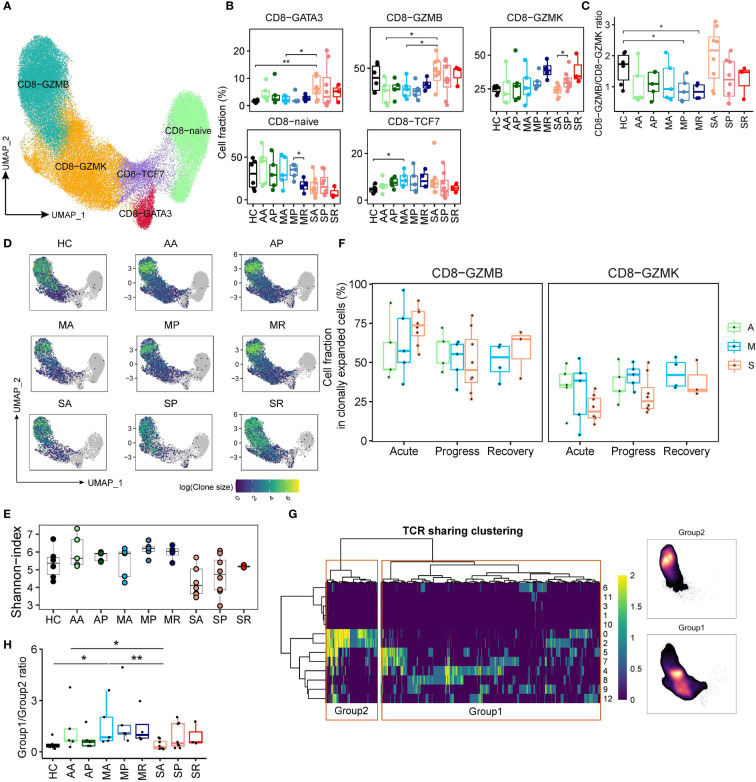
CD8^+^ T cell compartments respond differently in patients with severe COVID-19 versus those with non-severe diseases. **(A)** UMAP plot of the peripheral CD8^+^ T cell subsets. **(B)** Proportions of peripheral CD8^+^ T cell subsets from COVID-19 patients and controls (two-sided Student’s t-test, *P < 0.05, **P < 0.01). **(C)** The plot indicates the ratio of CD8-GZMB/CD8-GZMK from each studied group. **(D)** UMAP projection of clonally expanded CD8^+^ T cells from each studied group. **(E)** Shannon-index of total CD8^+^ T cell from each studied group. **(F)** The proportions of GZMB-CD8 and GZMK-CD8 subsets within the clonally expanded CD8^+^ T cell compartments. A, Asymptomatic; M, Mild; S, Severe. **(G)** TCR clustering analysis. Hierarchical clustering of TCRs (columns) based on TCR sharing patterns across CD8^+^ T subsets (rows). The two distinct groups identified are indicated in left box (group 2) and right box (group 1) (left). UMAP projection of cell density from TCR-group 1 and group 2 CD8^+^ T cells (Right). **(H)** The ratio of CD8^+^ T cells containing TCRs from group1 over cells containing TCRs from group 2 among each studied group (*P < 0.05, **P < 0.01).

Next, we identified 5 clusters of peripheral CD8^+^ T cells, as the CD8-CCR7 (Naïve), CD8-TCF7 (central memory), CD8-GATA3, CD8-GZMK (effector memory), and CD8-GZMB (terminal differentiated effector memory) subsets based on well-studied markers (**Figures 4A** and **S4A**). The expression pattern of transcription factors (**Figure S4B**) demonstrates that CD8-GZMB strongly expresses the transcription factor *TBX21*, *PRDM1*, and *ID2*, while transcription factors *EOMES* and *BCL6* are more expressed in CD8-GZMK cells, suggesting the accuracy of CD8^+^ T cell clustering. The diminished number of Naïve CD8^+^ T cells in severe (SA, SP, and SR) COVID-19 patients (likely related to their old age), is clearly reflected by the UMAP projections (**Figure S4C**). We also found that average percentage of the peculiar CD8-GATA3 subset in SA was the highest among all studied groups (**Figure 4B** and **Figures S4D, E**). GATA3 has been reported highly expressed in peripheral CD8^+^ T cell from patients with systemic sclerosis, and functionally related to IL13 induction. Thus, CD8-GATA3-IL13 expression have been proposed to play roles in amplifying inflammation and regarded as a highly relevant biomarker for inflammatory diseases (26). Consistently, CD8-GATA3 in the SA group produced the highest levels of IL13 (**Figures S4F**). Within the memory and effector CD8^+^ T cell compartment, we observed a discordance of CD8-GZMK and CD8-GZMB subset in SA (with a dominance of CD8-GZMB over CD8-GZMK) compared with other COVID-19 groups (**Figure 4C**). This was also robustly confirmed in RNA-seq data set (**Figure S4G**).

Next, we studied cycling CD8^+^ T cells and traced clonal status using the single-cell TCR (sc-TCR) data. Consistent with viral infection triggering immune response, the frequencies of cycling immune cells, and cycling CD4^+^ and CD8^+^ T cells were increased among COVID-19 patients compared to controls (**Figures 2B** and **S4H**). Then, we utilized UMAP projection to overview the TCR status, and confirmed that clonally expanded populations were mainly composed of CD8-GZMB and CD8-GZMK subsets (**Figures 4D** and **S4I, J**). Using the Shannon-index to reflect diversity, we found that patients with severe COVID-19 compared to those of other groups had lower levels of TCR diversity (**Figure 4E**). Moreover, within the clonally expanded CD8^+^ T cell compartment, a similar discordance of CD8-GZMK and CD8-GZMB subset was observed in COVID-19 patients. We found that increased proportion of clonally expanded CD8-GZMK seems to closely correlate with successful control of the SARS-CoV-2 infections, as early increase of CD8-GZMK in asymptomatic (AA/34.6% and AP/35.9%) and mildly ill (MA/31.0%, MP/41.3%, and MR/42.9%) cases, versus delayed increase of CD8-GZMK in severely ill patients (SA/20.8%, SP/29.4%, and SR/38.1%) (**Figures 4F** and **S4J**), indicating clonally expanded CD8-GZMK may play a role in viral clearance. Moreover, the percentage of CD8-GZMK cells sharing TCRs between sequential samples were higher in AA-AP and MA-MP transition than that in SA-SP (**Figure S4K**), also supporting that the clonally expanded the anti-viral CD8-GZMK population was established earlier in asymptomatic and mild cases than in severe cases.

Furthermore, integrating the scTCR-seq and scRNA-seq datasets using hierarchical clustering revealed one set of TCRs (group 1) with the cytotoxic phenotype and another set of TCRs (group 2) within the memory phenotype (**Figure 4G**). The proportions of group 1 and group 2 CD8^+^ T cells were varied among COVID-19 groups (**Figure S4L**), with higher percentage of group 1 cells in patients with severe COVID-19. The ratio of group 1/group 2 from SA is significantly lower than that from the AA and MA (**Figure 4H**), suggesting that the dominance of group 1 CD8^+^ T cells at the early stage of infection was associated with worse outcomes. Thus, our data suggest that the CD8-GZMK subset, as the group 2 CD8^+^ T cell equivalent, likely contains the majority of virus-responding T cells, and helps determine COVID-19 outcomes.

### Peripheral CD4^+^ T Cell Compartments and the Development of Severe or Non-Severe COVID-19

**Figure 5:**
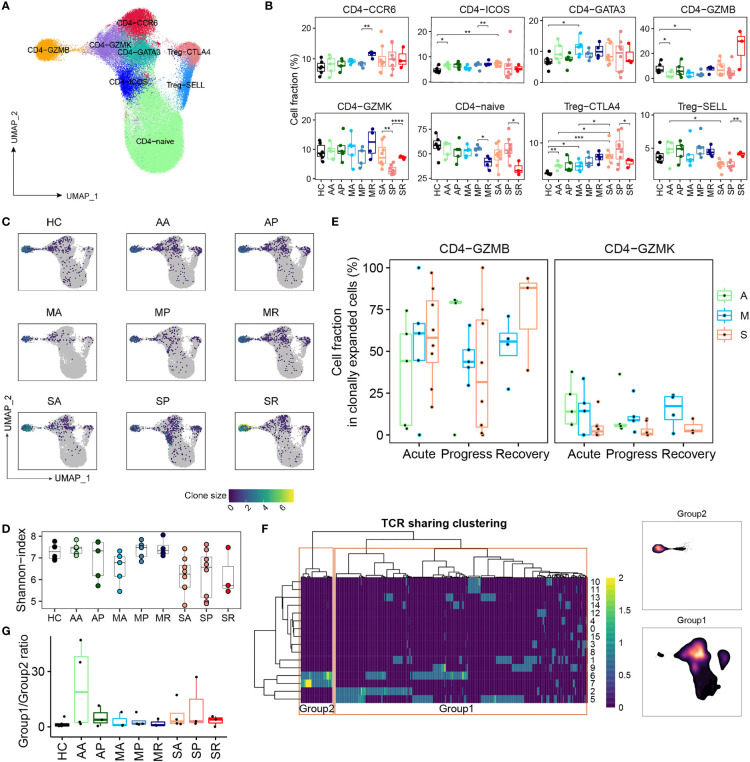
The peripheral CD4^+^ T cell compartment and its association with COVID-19 severity. **(A)** UMAP plot of the peripheral CD4^+^ T cell subsets. **(B)** Proportions of peripheral CD4^+^ T cell subsets from each studied group (two-sided Student’s t-test, *P <0.05, **P <0.01, ***p <0.001, ****P <0.0001). **(C)** UMAP plot of clonally expanded CD4^+^ T cells from each studied group. **(D)** The Shannon-index of total CD4^+^ T cells from each studied group. **(E)** The proportions of GZMB-CD4 and GZMK-CD4 subsets within the clonally expanded CD4^+^ T cell compartments. A, Asymptomatic; M, Mild; S, Severe. **(F)** TCR clustering analysis. Hierarchical clustering of TCRs (columns) based on TCR sharing patterns across CD4^+^ T subsets (rows). The two distinct groups identified are indicated in left box (group 2) and right box (group 1) (left). UMAP projection of cell density from TCR-group 1 and group 2 CD4^+^ T cells (right). **(G)** The ratio of CD4^+^ T cells containing TCRs from group 1 over cells containing TCRs from group 2 among each studied group.

We identified eight subpopulations of CD4^+^ T cells, namely, CD4-Naïve (*SELL*), Tfh-like (CD4-ICOS), Th1-like (CD4-GZMK), Th2-like (CD4-*GATA3*), Th17-like (CD4-*CCR6*), Treg-SELL and Treg-CTLA4 (*FOXP3*), cytotoxic phenotype (CD4-*GZMB*) (**Figures 5A** and **S5A**). Density UMAP plots revealed the increase of non-Naive cells as one obvious perturbation of peripheral CD4^+^ T compartments by the COVID-19 (**Figure S5B**). Notably, the percentage of Treg-CTLA4 cells increased significantly in most COVID-19 groups over controls, but the proportions of other CD4^+^ T cell subsets did not change significantly (**Figure 5B**). Among COVID-19 patients, we observed the trend of increased Treg-CTLA4 and CD4-GZMB, and decreased CD4-Naïve and CD4-GZMK in severe over non-severe groups (**Figures 5B** and **S5C, D**). The signature of T follicular helper (Tfh) (*IL21* in the CD4-ICOS cluster) and the signature of Th17 (*IL22* in the CD4-CCR6 cluster) tend to increase in SP patients, reflecting a dysregulated immune firing, while the polarization of the response of other T helper cells was not obvious (**Figure S5E**). Except for CD4-GZMB, the remaining CD4^+^ T cell subsets manifested lower levels of clonal expansion (**Figures 5C** and **S5C, D**). The severe COVID-19 patients had the lowest diversity of CD4^+^ T cell clonotypes among all studied groups (**Figure 5D**). Within the clonally expanded CD4^+^ T cell compartment, we observed overall decreased CD4-GZMB and increased Th1-, Th2-, Th17-, and cycling CD4^+^ T cell proportions from COVID-19 patients versus controls (**Figures S5F, G**). Notably, excluding CD4-GMZB, the Th1-like CD4-GZMK subset dominated in the expanded CD4^+^ T cell compartment in non-severe COVID-19 cases, but only represented a minor subset in severe cases (**Figures 5E** and **S5D**), indicating a discordant CD4^+^ T cell responses likely underlying the development of severe COVID-19. Moreover, percentage of TCR-sharing Th1-like (CD4-GZMK) cells between sequential samples were higher in AA-AP and MA-MP transition than that in SA-SP (**Figure S5H**), also supporting that clonally expanded CD4-GZMK cells were established earlier in non-severe cases, and likely played an important role in viral clearance.

Next, we integrated the scTCR-seq and scRNA-seq datasets of CD4^+^ T cells by hierarchical clustering and revealed one set of TCRs (group 1) showing the mix phenotype including the Th1-, Th2-, and Th17 subset and a group 2 within the cytotoxic phenotype (**Figure 5F**). Except that group 1 CD4^+^ T cells were enriched in SR, there was very little CD4^+^ T cell clonal expansion in the remaining 8 groups (**Figure S5I**). The ratio of group 1 and group 2 was also very small in 9 groups, making it hard to tell any differences (**Figure 5G**).

### Characterization of B Cell Subsets in COVID-19 Patients

**Figure 6:**
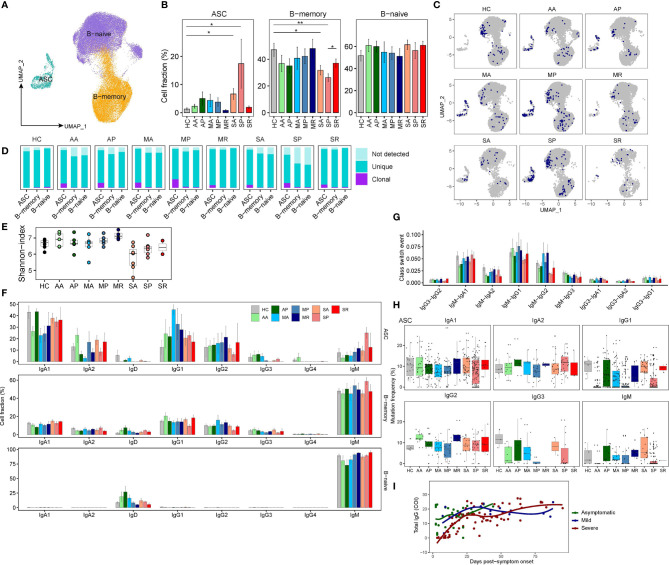
The peripheral B cell heterogeneity and its association with COVID-19 severity. **(A)** UMAP plot peripheral B cell subsets. **(B)** Proportions of peripheral B cell subsets from each studied group (two-sided Student’s t-test, *P <0.05, **P <0.01). **(C)** UMAP plot of clonally expanded B cells from each studied group. **(D)** Bar plots show the levels of clonal expansion within each B cell subsets from each studied group. **(E)** The Shannon-index of total B cells from each studied group. **(F)** Bar plots showing the proportions of cells with corresponding Ig isotypes within three B cell subsets from each studied group. **(G)** Bar plots show the class-switched ratio between different Ig isotypes across nine groups. **(H)** Bar plots show the frequency of somatic mutations of ASC with different Ig isotypes across nine groups. **(I)** The scatter plot shows the dynamics of RBD antibodies in the plasma of those 18 patients.

B cells were subclustered into three subsets by canonical markers, NAMELY, naïve B cells (*TCL1A*), memory B cells (MBC) (*CD27*) and Antibody secreting cells (ASC) (*MZB1*) (**Figures 6A** and **S6A**). Density UMAP plots clearly show that the proportion of ASCs from COVID-19 patients increases from the acute to the progressive infection stage, then subsides during the recovery stage (**Figure S6B**). We also found significantly decreased percentages of MBC in SA and SP compared to controls (**Figures 6B** and **S6C, D**). Since the proportion of ASC in patient S2 is abnormally high due to the presence of one massively expanded clone (**Figures S6E, F**), we excluded data from S2 in the following analysis.

Next, we analyzed scBCR-seq data. Notably, ASC in COVID-19 patients were more clonally expanded, consistent with the increased frequency of this population in the response to infection, whereas other B cells were non-expanded (**Figures 6C, D**). Compared to controls, asymptomatic and mildly ill patients, severely sick COVID-19 patients showed lower BCR diversity (**Figure 6E**). We examined proportions of each immunoglobulin (Ig) heavy chain isotypes within the different B cell subsets (**Figure 6F**). Indeed, naive B cells contained only IgD/IgM, while memory B cells and ASCs contained class-switched isotypes, namely, IgA and IgG. IgM presents a major fraction in naïve and memory B cells from all studied groups, while the IgG1 and IgA1 accounts for the majority of Ig isotypes in ASC. Consistent with reports showing IgG1 as the major responding Ig isotype during SARS-CoV-2 infection, the proportion of IgG1 isotype in ASC is increased among COVID-19 patients (**Figure 6F**). We observed significantly enriched IgM-IgA1, IgM-IgG1, and IgM-IgG2 events in most patients, especially in asymptomatic and mildly ill patients (**Figure 6G**). Previous studies have shown that SARS-CoV-2 targeting antibodies exhibit limited somatic hypermutation (SHM) (27). Since ASC is the antibody-producing population and more clonally expanded, we evaluated their SHM levels among each Ig isotypes. Indeed, SHM levels were higher in class-switched isotypes (both IgAs and IgGs) than non-class-switched IgM isotype (**Figure 6H**). However, we observed lower levels of SHM (even germline without SHM) in ASCs from COVID-19 patients, especially in the IgG1 isotype. Interestingly, the timing of germline IgG1 ASCs emergence differed in asymptomatic, mildly ill patients and severely sick patients. Those unusual ASCs, likely the virus responding ones, emerged earlier in AA and MA, and emerged later in SP. Similar trends were also present in IgG3 and IgM isotype (**Figure 6H**). We suspect that our data reveal a delayed antibody response in patients destined to become severely ill. Indeed, we found higher levels of serum SARS-CoV-2-Spike-RBD (Receptor binding domain on Spike protein)-specific antibodies in the blood of AA and MA compared to SA patients (**Figure 6I**), and made similar observations on a larger cohort of 506 COVID-19 patients (28). Thus, although many reports showed higher levels SARS-CoV-2 antibodies in patients with severe COVID-19 than those in mild COVID-19 (29), the humoral immune defense may actually be initiated earlier in patients with mild disease.

## Discussion

For severe COVID-19, the entire clinical course is dynamic and includes asymptomatic, symptomatic, ARDS and recovery phases (1). Thus, patients with severe COVID-19 would manifest very different symptoms, and very likely distinct immune responses at those different stages of the infection or diseases. Understanding the pathogenic mechanisms in the deterioration and recovery of severe COVID-19 will require a complete monitoring the entire dynamic immune responses.

Many efforts have been attempted to dissect the heterogeneous immune responses in patients with different clinical manifestations, namely, multiple studies using high-throughput single-cell approaches (30–33). However, most of these previous reported datasets which investigated cross-sectional samples collected at peak level severity or convalescence. As a result, there is a lack of datasets for longitudinal samples collected from an earlier stage of infection. So far, little is known about the early immunological events that could affect the development of severe versus asymptomatic/mild diseases. In addition, previous immune investigations comparing samples collected at unsynchronized clinical phases could lead to inconsistent conclusions (18, 19). In contrast, we provide a valuable scRNA-seq analysis of peripheral immune cells in patients infected with SARS-CoV-2, covering longitudinal specimens of COVID-19 patients with asymptomatic, mild, and severe diseases collected shortly after the symptom onset. Indeed, our data reveal a highly dynamic immune landscape particularly in severe COVID-19, matching with the different stages (Acute, Progression, and Recovery) of clinical courses, whereas the composition of immune cellular compartment in patients with asymptomatic/mild COVID-19 is rather stable. Particularly, the data that we describe here provide a useful resource for precisely deciphering the early immunological events preceding the worsen or resolution of SARS-CoV-2 induced disease.

IFN response is the first line of host innate immune defense against viral infection. We and others have previously reported that IFN response is impaired in patients with severe COVID-19 based on cross-sectional samples (10, 24). But here, we were surprised to find that severe patients had a transient strong IFN response before the disease deterioration, but then dropped rapidly. While the mild patients have a weak but stable interferon response throughout the disease. High concentrations of IFN in plasma of SA patients are most likely stimulated by viral infections. SARS-CoV-2 infection can induce strong IFN production and is positively correlated with viral load (23). In the absence of animal models that can mimic severe COVID-19, it is difficult to determine whether IFNs serve a protective or a detrimental function in COVID-19. There are some studies reporting the pathological role of IFN during severe coronavirus infections (7, 34, 35). Hospitalized COVID-19 patients with high levels of pulmonary ISGs died significantly earlier than those with low levels of ISGs in a transcriptomic study of the lung samples (35). Another study found that IFN played inflammatory roles by recruiting more immune cells to the lungs (34). It has been reported that IFN disrupt lung epithelial repair and the pulmonary epithelial barrier upon viral recognition (36, 37). In addition, IFN can disrupt the urea cycle, reducing arginine levels and thus dampening the T cell functions (38). Arginine in plasma in patients with severe COVID-19 is indeed lower than in mild cases (39). Based on these data, we propose that a strong early transient IFN response may aggravate the progression of COVID-19, by impairing T-cell responses.

Regarding protective anti-viral adaptive immune components, CD8^+^ T cells are a unique immune cell population that could precisely and efficiently clear virus-infected host cells. Although SARS-CoV-2 reacting T cells responses were detected in COVID-19 patients (14, 40–42), and their roles in determining disease severity are postulated, so far, their roles have not been definitively defined. Here, our data revealed that the CD8^+^ T cell likely played a crucial role in controlling SARS-CoV-2 infection, especially in the early stages of infection. We found the lack of early induction of CD8^+^ T cell responses as a prominent feature of severe COVID-19. In agreement with other recent reports (13, 43), this impaired induction of CD8^+^ T cell responses in severe COVID-19 was likely a result of decreased numbers of naive CD8^+^ T cells. This could also explain why old age is an important risk factor for development of severe COVID-19. Old adults are known to have a lower number of naive CD8^+^ T cells, and therefore they are less likely to be effective responders to handle new viral pathogens.

In addition, our data demonstrated the importance of humoral immune defense in SARS-CoV-2 infection. Interestingly, sequencing the BCR repertoire showed that the early recruitment of B cells with low SHMs signatures was associated with seroconversion of SARS-CoV-2 IgG (44). It was later reported that antibodies against the SARS-CoV-2 spike protein receptor binding domain (RBD) are primarily mediated by the near-germline IgG1 antibodies with low levels of SHMs (27). Thus, the appearance of low SHM IgG1 sequences in ASCs observed in this study, likely indicates the antibody response to SARS-CoV-2 RBD. Importantly, both the low SHM IgG1 signature and RBD antibodies occurred later in patients with severe COVID-19, suggesting delayed engagement of effective humoral immunity as another predictor for onset of severe disease. This is consistent with recent studies showing delayed neutralizing antibodies correlate with fatal COVID-19 (45, 46).

In conclusion, we provided convincing evidence that the early immunological events, namely, abnormal strong interferon response, delayed CD8^+^ T-cell engagement, and humoral immune responses, may determine the subsequent progression of severe COVID-19. Additionally, we provide a number of early prognostic markers for the onset of severe COVID-19, such as CD14^+^/CD16^+^ monocytes ratio, CD4^+^/CD8^+^T cell ratio, GZMK^+^/GZMB^+^ T cell ratio, etc., although these parameters require further validation in the larger cohorts.

## Materials and Methods

### Patients

Ethics statement: This study was conducted according to the ethical principles of the Declaration of Helsinki. Ethical approval was obtained from the Research Ethics Committee of Shenzhen Third People’s Hospital (2020-242).

All participants provided written informed consent for sample collection and subsequent analyses. Eighteen COVID-19 patients were enrolled at the Shenzhen Third People’s Hospital for scRNA-seq study. Samples from metadata and patients were collected similarly as previously described: The severity of COVID-19 was categorized to be mild, moderate, severe and critical according to the “Diagnosis and Treatment Protocol of COVID-19 (the 7th Tentative Version)” by the National Health Commission of China (http://www.nhc.gov.cn/yzygj/s7653p/202003/46c9294a7dfe4cef80dc7f5912eb1989.shtml). In this study, we grouped patients with mild and moderate COVID-19 as the mild group, and included those with severe and critical diseases as the severe group. Asymptomatic patients have no clinical symptoms such as cough or fever within 1–2 weeks from a positive nucleic acid test to negative. Six healthy subjects were enrolled as the control group.

### Blood Samples Process

Approximately 5–10 ml of fresh blood is separated into plasma after centrifugation, which will be used for cytokine detection later. The remaining cells underwent Ficoll–Hypaque density gradient centrifugation to obtain PBMC, which can be used for single cell sequencing.

### Cytokines Measurement by MSD

Plasma from 10 severe patients, 9 mild patients, and 11 asymptomatic patients was used for cytokine measurement. Twelve healthy subjects were enrolled as the control group. IFNγ, IFN-α2a, and IL-29/IFN-λ1 were detected according to the instruction (MESO SCALE DISCOVERY, K15067L-1). In brief, 25 ul samples or standards were incubated in antibody coupled plate at room temperature for 1h, detection antibodies were added for 1 h after washing by PBST. Finally, MSD GOLDTM Read Buffer B was added to read the results.

### Detection of Plasma Antibodies

The plasma of 18 patients in the acute phase in this study were collected, and chemiluminescence kit (Beijing Wantai Biotech) in the Caris200 automatic chemiluminescence instrument was used to detect the level of IgG antibody against SARS-CoV-2-Spike-RBD. The relative fluorescence of sample to control (COI) was used to estimate the result. The results ≥1 COI are reactive (positive), and the results <1 COI are nonreactive (negative).

### ScRNA-Seq Library Construction

ScRNA-seq libraries were prepared according to previous protocols. In brief, the recovered PBMC were counted in 0.4% trypan blued, centrifuged and re-suspended at the concentration of 2 × 10^6^/ml. The cell suspension was loaded onto a Chromium single cell controller (10× Genomics) to generate single-cell gel beads in the emulsion (GEMs) according to the manufacturer’s protocol. Reverse transcription takes place inside each GEM, after which cDNAs are pooled together for amplification and library construction. The resulting library products consist of Illumina adapters and sample indices, allowing pooling and sequencing of multiple libraries on the next-generation short read sequencer.

### Single Cell Filtering, Clustering, Dimension Reduction, and Visualization

We aligned the sequenced reads against GRCh38 human reference genome by Cell Ranger (version 3.1.0, 10× genomics). The raw count matrix (UMI counts per gene per cell) was processed by Seurat (v3.2.2) (47). Cells with less than 200 and more than 6,000 expressed genes, less than 1,000 UMI and higher than 15% mitochondrial genome transcript were removed. Genes expressed in less than 3 cells were removed.

Data integration, cell clustering and dimension reduction were performed by Seurat (v3.2.2). First, the gene expression matrix were normalized using the “NormalizeData” function with default settings. The sources of cell–cell variation driven by batch were regressed out using the number of detected UMI and mitochondrial gene expression, which were implemented using the “ScaleData” function. The top 2,000 highly variable genes (HVGs) were used for the following analysis using “FindVariableFeatures” function. Next, we integrated different samples by “IntegrateData” function, which eliminates technical or batch effect by canonical correlation analysis (CCA). Using those HVGs, we calculate a PCA matrix with the top 50 components by “RunPCA” function. The cells were then clustered by “FindClusters” function after building nearest neighbor graph using “FindNeighbors” function. The parameter resolution was set to 0.4 to identify cell types in all cell populations. The cluster-specific marker genes were identified by “FindMarkers” function using MAST algorithm (v1.15.0). The clustered cells were then projected into a two-dimension space for visualization by a non-linear dimensional reduction method “RunUMAP” in Seurat package.

### Integrated Analysis of Peripheral Myeloid, CD4^+^ T, CD8^+^ T, Innate T and B Cells

We re-clustered the peripheral myeloid, CD4^+^ T, CD8^+^ T, innate T, and B cells using the top 20 dimensions of PCA with the parameter resolution of 0.6, 1.3, 1.3, 0.8, and 1.3 respectively. The myeloid compartment, namely, mDCs and monocytes was re-clustered using cells annotated with monocyte and mDCs in **Figure 2**. The CD4^+^ T and CD8^+^ T cells were re-clustered using cells annotated with CD4^+^ and CD8^+^ T cells in **Figure S2**. The B cell subsets were re-clustered using B cells annotated in **Figure 2**. The re-clustered cells were annotated by canonical markers.

### Differentially Expressed Gene and Gene Enrichment Analysis

The “FindMarkers” function in Seurat with MAST algorithm (v1.15.0) (48) was used to analyze DEGs. For each pairwise comparison, the “FindMarkers” function was run with the parameters of test.use = ‘MAST’. Genes were defined as significantly upregulated if the average natural logarithm fold change (logFC) was >0.25 and adjusted P-value was <0.01. The genes with logFC <−0.25 and adjusted P <0.01 were considered significantly downregulated. We performed GO term enrichment analysis for the significantly upregulated and downregulated genes using clusterProfiler (v3.17.3) (49) package in R (v4.0.2). GO term of Biological Process (BP) was displayed.

### Principal Component Analysis of All Samples

The principal component analysis of all samples in **Figure 1** was calculated using the average expression level of the top 4,000 HVGs across all cells in each sample utilizing “prcomp” method in R (v4.0.2).

### Calculation of Immune Signature Scores

Immune signature scores in scRNA-seq data were calculated using the AddModuleScore function in the Seurat package. IFN response scores were calculated using *ADAR, APOBEC3, BST2, CD74, MB21D1, DDIT4, DDX58, DDX60, EIF2AK2, GBP1, GBP2, HPSE, IFI44L, IFI6, IFIH1, IFIT1, IRF1, IRF7, ISG15, ISG20, MAP3K14, MOV10, MS4A4A, MX1, MX2, NAMPT, NT5C3, OAS1, OAS2, OAS3, OASL, P2RY6, PHF15, PML, RSAD2, RTP4, SLC15A3, SLC25A28, SSBP3, TREX1, TRIM5, TRIM25, SUN2, ZC3HAV1, IFITM1, IFITM2*, and *IFITM3*. The MHC class II score was calculated using *HLA-DMA, HLA-DMB, HLA-DPA1, HLA-DPB1, HLA-DQA1, HLA-DQB1, HLA-DRA, HLA-DRB1*, and *HLA-DRB5*. The alarmin score was calculated using *S100A1, S100A2, S100A3, S100A4, S100A5, S100A6, S100A7, S100A7A, S100A7L2, S100A7P1, S100A7P2, S100A8, S100A9, S100A10, S100A11, S100A12, S100A13, S100A14, S100A15A, S100A16, S100B, S100G, S100P*, and *S100Z*. “Monocyte composite scores” were calculated according to the MHC-II score minus alarmin score. The cytotoxicity score was calculated using *PRF1, IFNG, GNLY, NKG7, GZMB, GZMA, GZMH, KLRK1, KLRB1, KLRD1, CTSW*, and *CST7.*

The ISGs score, MHC II score and alarmin score in a sample with bulk RNA-seq data were calculated as the geometric mean of the normalized log2-transformed expression of the genes above separately.

### Estimation of Cell Composition in Bulk RNA-Seq Data

We used MarkerBasedDecomposition function in Bisque, a semi-supervised model that extracts trends in cellular composition from normalized bulk expression samples, to deduce cell type abundance using only cell-specific marker genes: CD79A, CD19, MS4A1 marked B cells. CD79A and IGKC marked PCs. CD3D, CD4 and GZMB marked CD4-GZMB. CD3D, CD4 and GZMK marked CD4-GZMK. CD3D, CD4 and FOXP3 marked CD4-Treg. CD3D, CD8A and GZMB marked CD8-GZMB. CD3D, CD8A and GZMK marked CD8-GZMK. FCN1 and CD14 marked mono-CD14+. FCN1 and FCGR3A marked mono-CD16+.

### Single-Cell TCR and BCR Analysis

The amino acid and nucleotide sequence of TCR/BCR chains were assembled and annotated by cellranger vdj function in CellRanger (version 3.1.0). For TCR, only cells with paired TCRα and TCRβ chains were included in clonotype analysis. Cells sharing the same TCRα- and TCRβ-CDR3 amino acid sequences were assigned to the same TCR clonotype. For the BCR, only cells with at least one productive heavy chain (IGH) and one productive light chain (IGK or IGL) were kept for further analysis. Cells sharing the same V/J gene and the same IGH- and IGK/IGL-CDR3 amino acid was defined as a clonotype. The TCR clonotypes and the BCR clonotypes were integrated into transcriptome object using barcode information. Shannon index (TCR/BCR diversity) of each sample was calculated using “diversity” function in vegan package (v2.5.6) (https://github.com/vegandevs/vegan) in R. For each BCR, we calculated their similarity to the germline genes using the V gene on heavy chain utilizing IgBlast (v1.15.0) (50). The SHM was deduced using the difference between 1 and the above calculated similarity.

TCR sharing clustering analysis. Referring to the methods from a recent report (19), we constructed a TCR matrix of CD4^+^ and CD8^+^ T cells with cell cluster as rows and unique TCRs as columns with the number of cells with a given TCR in a certain cluster as values. Only the TCRs present in at least two clusters were kept for further analysis. The TCR matric were transformed *via* log1p transformation (formula = ln (value + 1)) and values were clipped at 2 (any value greater than 2 was set to 2). Both TCRs and cell clusters were subject to hierarchal clustering with the method set to “ward” using “pheatmap” function in R.

### RT-qPCR

All studies involving SARS-CoV-2 infection were conducted in the biosafety level-3 (BLS-3) laboratory of Shenzhen Third People’s Hospital. Lung epithelial cells Calu3 were infected with SARS-CoV-2 at 1 MOI for 24 and 48 h. Total RNA was extracted with TRIzolTM Reagent in accordance with the manufacturer’s instructions and reverse-transcribed into cDNA with a High-Capacity cDNA Reverse Transcription Kit (Takara, RR036A). The expression levels of indicated RNA were determined by RT-qPCR analysis using Power SYBR Green PCR Master Mix (Vazyme, Q311-02). Primers used in RT-qPCR reactions are listed in **Table S4**.

### Statistics

The Student’s t-test (t-test in R, two-sided, unadjusted for multiple comparisons) was used for pairwise comparisons of the cell proportions between different groups. The Pearson correlation coefficient between clinical index and WOS was evaluated utilizing the corr.test function in R (v4.0.2). The silhouette coefficient was calculated using the following formula:


$$\frac{b_{i}-a_{i}}{\max\{a_{i},b_{i}\}}$$

Where, *a_i_* indicates the mean of euclidean distance from cell i to all other cells that belong to the cell type. *b_i_* indicates the mean of euclidean distance from cell i to all other cells that is nearest to the cell type of i.

## Data Availability Statement

The raw data reported in this paper have been deposited in the Genome Sequence Archive in National Genomics Data Center, Beijing Institute of Genomics, Chinese Academy of Sciences under accession number(s) HRA000628 that are publicly accessible at http://bigd.big.ac.cn/gsa-human.

## Author Contributions

This study was conceived and designed by SZ and ZZ. GX conducted the major experiments. Bioinformatics analysis was performed by FQ and YaL. HW, YuL, XW, RZ, JY, and XL contributed some experimental data. The manuscript was written by ZZ, GX, SZ, and FQ. All authors contributed to the article and approved the submitted version.

## Funding

This study was supported by the National Science Fund for Distinguished Young Scholars (82025022), the Central Charity Fund of Chinese Academy of Medical Science (2020-PT310-009), the Shenzhen Bay Funding (2020B1111340075), the China National Natural Science Youth Foundation (82101857), the Guangdong Provincial Department of Science and Technology (2019A1515011072) and the Shenzhen Science and Technology Innovation Committee (KQTD20200909113758004). The funders had no role in study design, data collection, data analysis, data interpretation, or writing of the report.

## Conflict of Interest

The authors declare that the research was conducted in the absence of any commercial or financial relationships that could be construed as a potential conflict of interest.

## Publisher’s Note

All claims expressed in this article are solely those of the authors and do not necessarily represent those of their affiliated organizations, or those of the publisher, the editors and the reviewers. Any product that may be evaluated in this article, or claim that may be made by its manufacturer, is not guaranteed or endorsed by the publisher.
