# Supplementary material for: The Transient IFN Response and the Delay of Adaptive Immunity Feature the Severity of COVID-19
Source: Front Immunol. 2022 Jan 14;12:816745. doi: 10.3389/fimmu.2021.816745 (PMC8795972; doi:10.3389/fimmu.2021.816745)
Supplement: Supplementary file 1 [file DataSheet_1.docx]

Supplementary Material

## Supplementary Figures


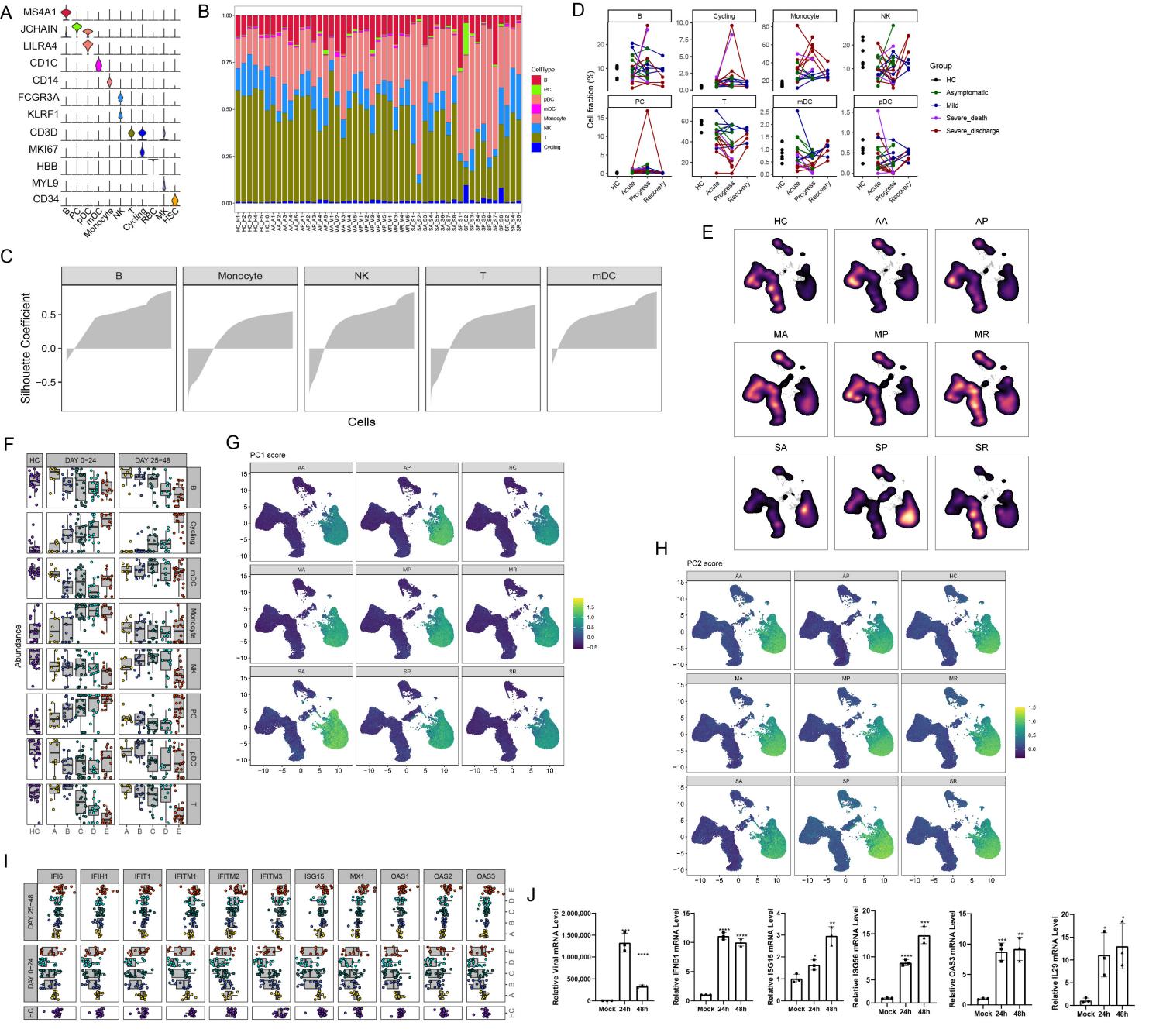


**Supplementary Figure 1.** **Data related to Fig. 2.**

(A) Specific markers for identifying cell types in (Fig 2A).

(B) Bar plots shows peripheral immune cell heterogeneity from each sample.

(C) The Silhouette coefficient of each cell type is uniformly distributed, with an average silhouette coefficient of 0.48.

(D) The dynamic changes of the peripheral immune cells of each COVID-19 patient at 3 different time points.

(E) UMAP projections of cell density from COVID-19 patients and control.

(F) The scatter plot shows the percentage of the eight immune cell subsets identified in Fig. 2a of the 5 groups of COVID-19 patients and healthy controls based on the Marker Based Decomposition analysis of the RNA-seq data in the 2 time zones.

(G) UMAP plot of PC1 (from Fig. 2C) gene scores from each studied group.

(H) UMAP plot of PC2 (from Fig. 2C) gene scores from each studied group.

(I) The scatter plot shows the expression of 11 ISGs in the RNA-seq data of PBMC of 5 groups of COVID-19 patients with different severity levels and healthy controls at 2 time points.

(J)Bar plots show the expression of IFN and some ISGs in Calu3 cells 24 and 48 hours after SARS-CoV-2 infection. Three repeated experimental data were used for Paired t-test, (*, P < 0.05, **, P < 0.01, ***, P < 0.001 ****, P <0.0001.).


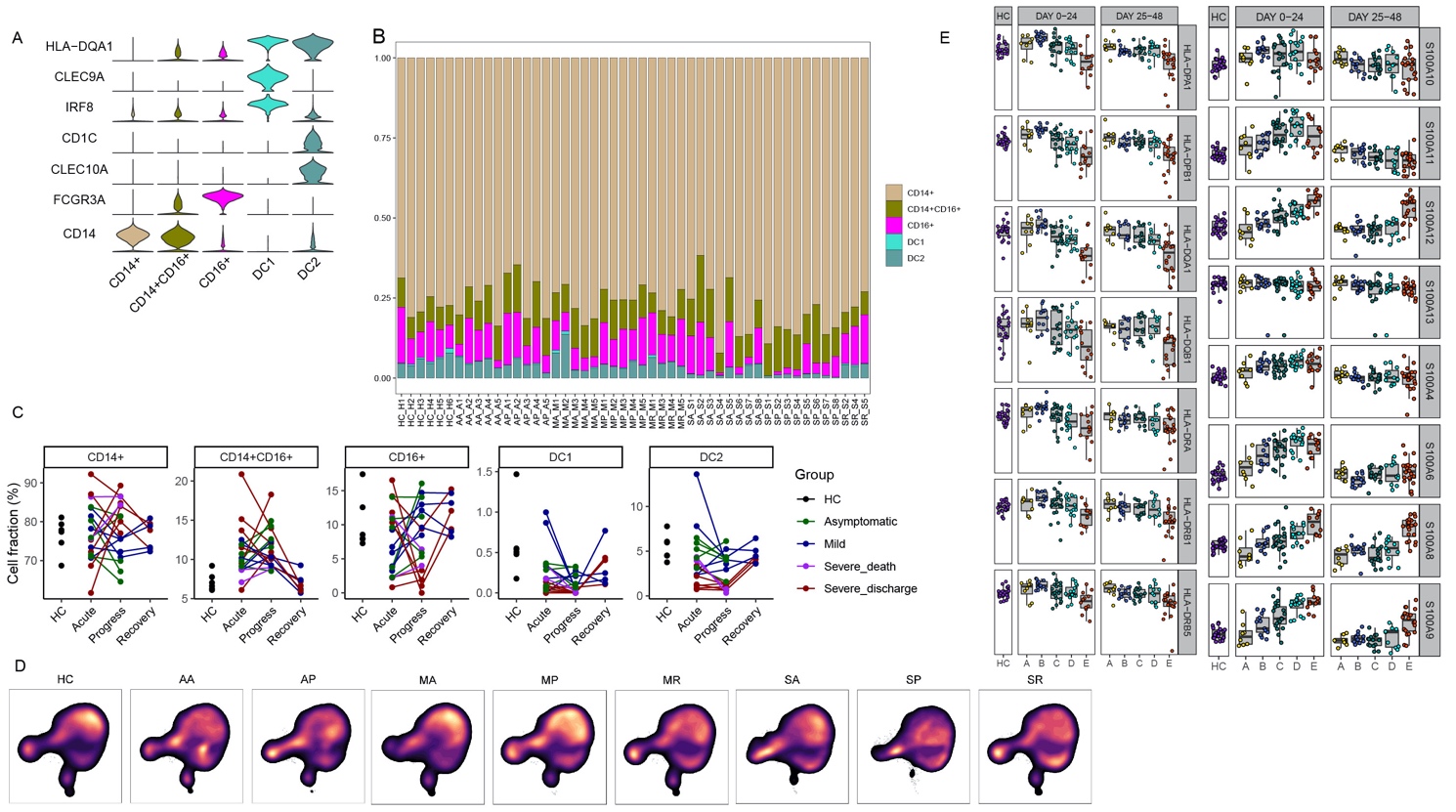


**Supplementary Figure 2.** **Data related to Fig. 3.**

(A) Violin plot of marker genes expression by different myeloid cell types.

(B) Bar plot shows peripheral myeloid cell heterogeneity from each sample.

(C) The dynamic changes of the five myeloid cell subsets of each COVID-19 patient at 3 different time points.

(D) UMAP projection of myeloid cell density from COVID-19 patients and control.

(E) The scatter plot shows the dynamic expression of eight MHC II molecules (left) and eight alarmin molecules (right) of the 5 groups of COVID-19 patients and healthy control in the 2 time zones.


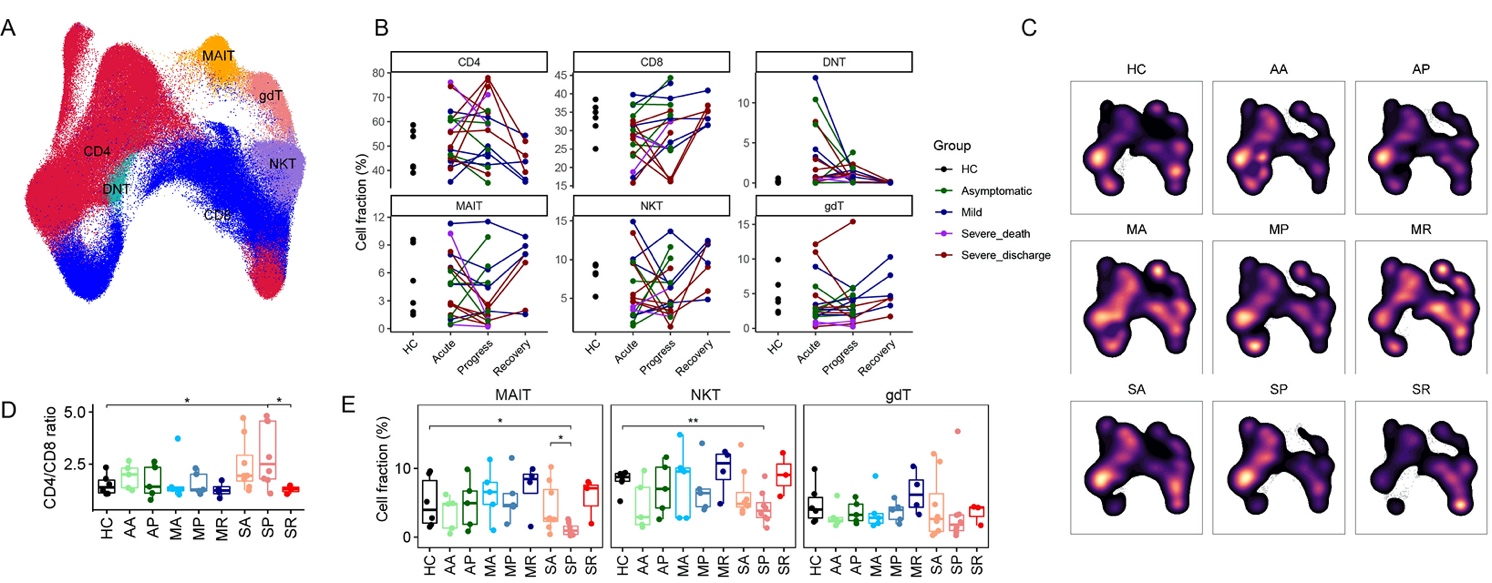


**Supplementary Figure 3.** **Characterization of peripheral T cell and innate-like T cell subsets.**

(A) UMAP plot of the peripheral T cell subsets.

(B) The dynamic changes of the six T cell subsets of each COVID-19 patient at 3 different time points.

(C) UMAP plot of T cell density from each studied group.

(D) The ratio of CD4/CD8 T cells from each studied group.

(E) Proportions of innate-like T cell subsets, including MAIT, NKT and γδ T cells from each studied group (two-sided Student’s t-test, *P < 0.05, **P < 0.01).


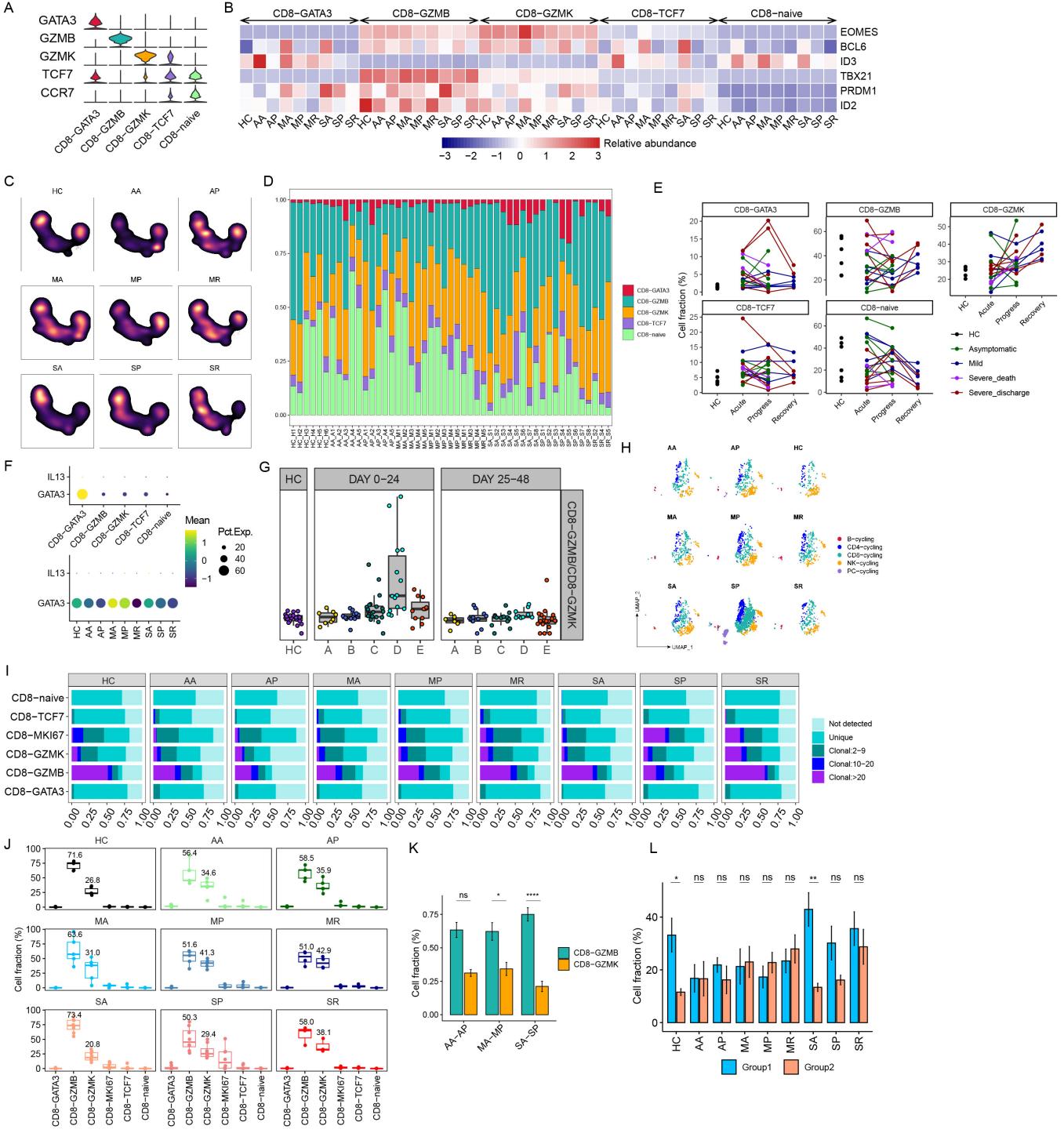


**Supplementary Figure 4. Data related to Fig. 4.**

(A) Expression of specific markers for identifying CD8^+^ T cell subsets.

(B) Expression of transcription factors related to CD8^+^ T cell subsets.

(C) UMAP projection of CD8^+^ T cell density from each studied group.

(D) Bar plot shows the five CD8^+^ T cell heterogeneity from each sample.

(E) The dynamic changes of the five CD8^+^ T cell subsets of each COVID-19 patient at 3 different time points.

(F) Expression of IL13 and GATA3 in different CD8^+^ T cell subsets (up panel) or different groups (bottom panel).

(G) The scatter plot shows the ratio of CD8-GZMB / CD8-GZMK of the 5 groups of COVID-19 patients and healthy control based on the Marker Based Decomposition analysis of the RNA-seq data in the 2 time zones.

(H) UMAP plot of peripheral cycling cells.

(I) Bar plot shows the levels of clonal expansion within each CD8^+^ T cell subsets from each studied group.

(J)The proportions of each CD8^+^ T cell subset within the clonally expanded CD8^+^ T cell compartments.

(K)Bar plot shows the proportions of cells sharing TCRs from CD8-GZMB or CD8-GZMK subset from asymptomatic, mild and severe COVID-19 patients.

(L)Bar plot shows the percentages of TCR-group1 and group2 CD8^+^ T cells from each studied group (* P < 0.05, ** P < 0.01, ns: not significant).


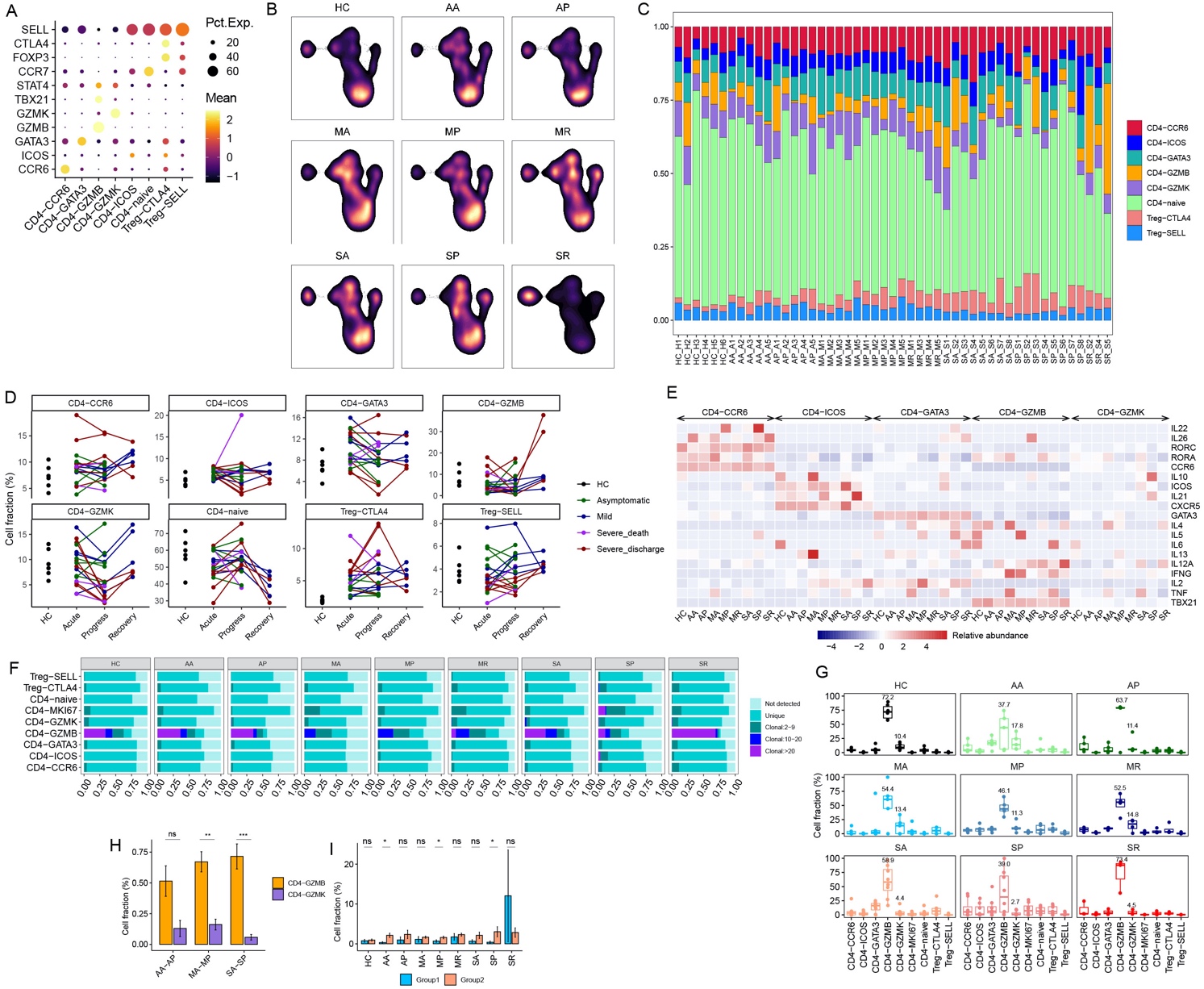


**Supplementary Figure 5. Data related to Fig. 5.**

(A) Expression of specific markers for identifying CD4^+^ T cell subsets.

(B) UMAP projection of CD4^+^ T cell density from each studied group.

(C) Bar plot shows the eight CD4^+^ T cell heterogeneity from each sample.

(D) The dynamic changes of the eight CD4^+^ T cell subsets of each COVID-19 patient at 3 different time points.

(E) Expression of specific markers associated with polarization of CD4 T cell subsets.

(F) Bar plot shows the levels of clonal expansion within each CD4^+^ T cell subset from each studied group.

(G) The proportions of each CD4^+^ T cell subset within the clonally expanded CD4^+^ T cell compartments.

(H) Bar plot shows the proportions of cells sharing TCRs from CD4-GZMB or CD4-GZMK subset from asymptomatic, mild and severe COVID-19 patients.

(I) Bar plot shows the percentages of TCR-group1 and group2 CD4^+^ T cells from each studied group.

**Supplementary Figure 6**
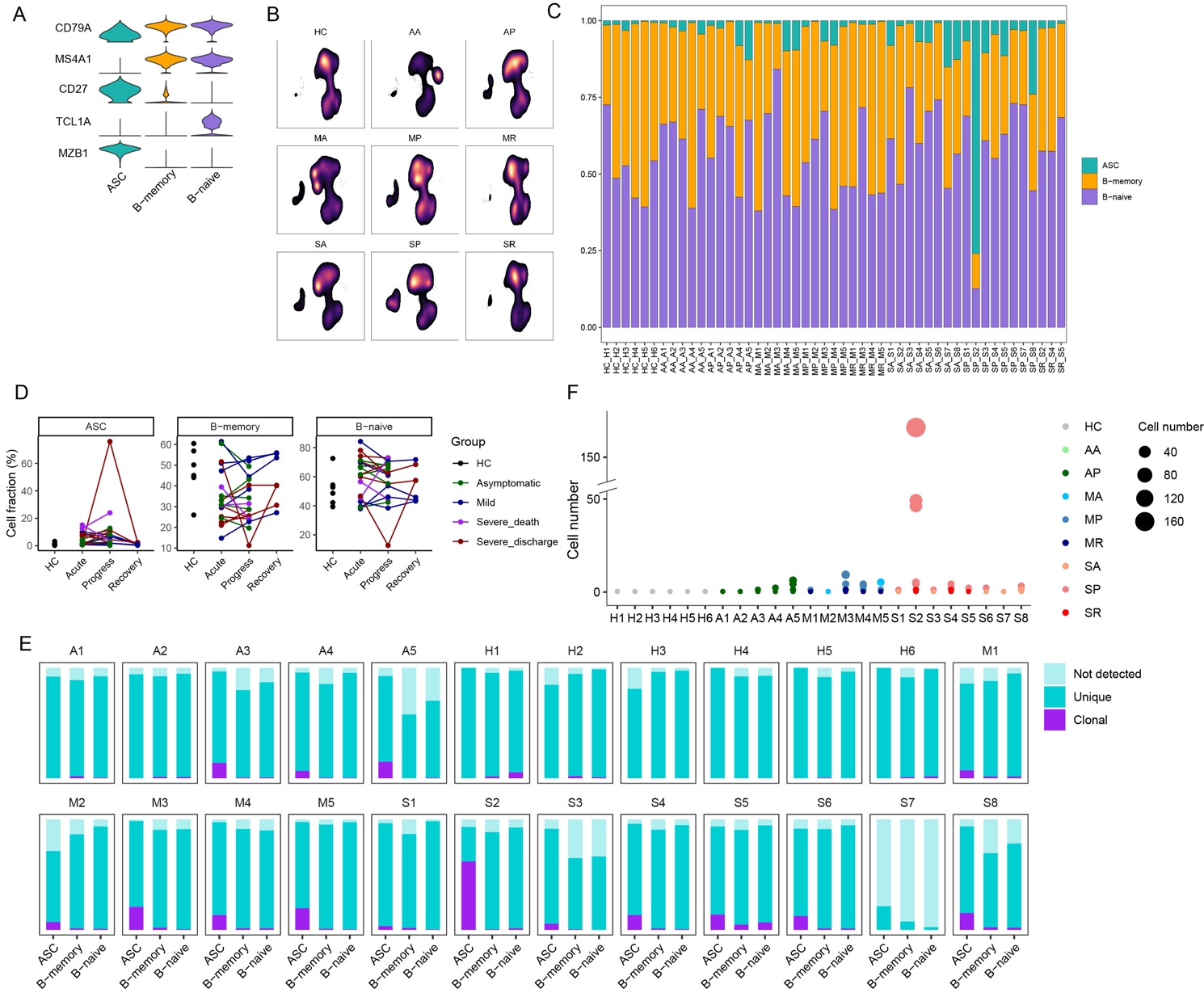
**. Data related to Fig. 6.**

(A) Specific markers for identifying B cell subsets in (Fig. 6A).

(B) UMAP plot of B cell density from COVID-19 patients and control.

(C) Bar plot shows the three B cell heterogeneity from each sample.

(D) The dynamic changes of the three B cell subsets of each COVID-19 patient at 3 different time points.

(E) Bar plot shows the levels of clonal expansion within each B cell subset from each patient.

(F) The dotplot represents the cell numbers in each expanded B cell clone from each sample.
